# 4-[(2,4-Dimethyl­thia­zol-5-yl)meth­yl]-4-hydr­oxy-2-methyl­isoquinoline-1,3(2*H*,4*H*)-dione

**DOI:** 10.1107/S1600536810010469

**Published:** 2010-03-27

**Authors:** Hoong-Kun Fun, Jia Hao Goh, Haitao Yu, Yan Zhang

**Affiliations:** a X-ray Crystallography Unit, School of Physics, Universiti Sains Malaysia, 11800 USM, Penang, Malaysia; b School of Chemistry and Chemical Engineering, Nanjing University, Nanjing 210093, People’s Republic of China

## Abstract

In the title isoquinoline­dione derivative, C_16_H_16_N_2_O_3_S, the piperidine ring in the tetra­hydro­isoquinoline ring system adopts a distorted envelope conformation. The thia­zole ring is essentially planar [maximum deviation = 0.004 (1) Å] and is inclined at a dihedral angle of 31.08 (3)° with respect to the mean plane through the tetra­hydro­isoquinoline ring system. In the crystal structure, inter­molecular O—H⋯O and C—H⋯O inter­actions link adjacent mol­ecules into a three-dimensional extended network. The crystal structure is further stabilized by weak C—H⋯π inter­actions.

## Related literature

For general background to and applications of isoquinoline­dione derivatives, see: Griesbeck *et al.* (2003 ▶); Hall *et al.* (1994 ▶); Malamas & Hohman (1994 ▶); Suau & Villatoro (1994 ▶); Zhang *et al.* (2004 ▶). For ring conformations, see: Cremer & Pople (1975 ▶). For related structures, see: Fun *et al.* (2010**a* ▶,b* ▶); Wang *et al.* (2000 ▶). For the stability of the temperature controller used for the data collection, see: Cosier & Glazer (1986 ▶).
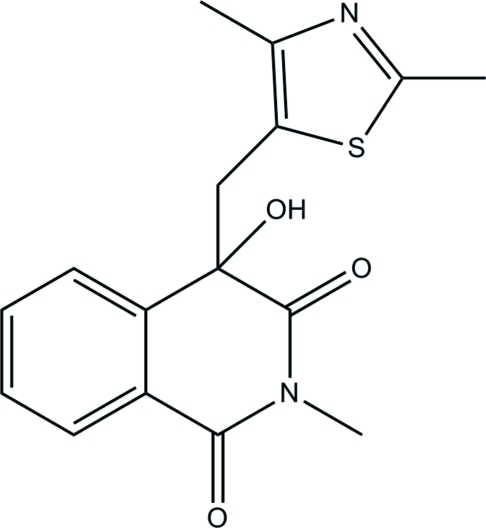


## Experimental

### 

#### Crystal data

C_16_H_16_N_2_O_3_S
*M* *_r_* = 316.37
Monoclinic, 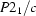

*a* = 10.2424 (8) Å
*b* = 15.0438 (13) Å
*c* = 9.4786 (8) Å
β = 92.839 (2)°
*V* = 1458.7 (2) Å^3^
*Z* = 4
Mo *K*α radiation
μ = 0.24 mm^−1^
*T* = 100 K
0.52 × 0.26 × 0.09 mm

#### Data collection

Bruker SMART APEX DUO CCD area-detector diffractometer
Absorption correction: multi-scan (*SADABS*; Bruker, 2009 ▶) *T*_min_ = 0.887, *T*_max_ = 0.979
20742 measured reflections
5260 independent reflections
4752 reflections with *I* > 2σ(*I*)
*R*_int_ = 0.024

#### Refinement

*R*[*F* ^2^ > 2σ(*F* ^2^)] = 0.031
*wR*(*F* ^2^) = 0.108
*S* = 1.14
5260 reflections
263 parameters
All H-atom parameters refined
Δρ_max_ = 0.69 e Å^−3^
Δρ_min_ = −0.47 e Å^−3^

### 

Data collection: *APEX2* (Bruker, 2009 ▶); cell refinement: *SAINT* (Bruker, 2009 ▶); data reduction: *SAINT*; program(s) used to solve structure: *SHELXTL* (Sheldrick, 2008 ▶); program(s) used to refine structure: *SHELXTL*; molecular graphics: *SHELXTL*; software used to prepare material for publication: *SHELXTL* and *PLATON* (Spek, 2009 ▶).

## Supplementary Material

Crystal structure: contains datablocks global, I. DOI: 10.1107/S1600536810010469/is2531sup1.cif

Structure factors: contains datablocks I. DOI: 10.1107/S1600536810010469/is2531Isup2.hkl

Additional supplementary materials:  crystallographic information; 3D view; checkCIF report

## Figures and Tables

**Table 1:** Hydrogen-bond geometry (Å, °) *Cg*1 is the centroid of C3–C8 benzene ring.

*D*—H⋯*A*	*D*—H	H⋯*A*	*D*⋯*A*	*D*—H⋯*A*
O3—H1*O*3⋯O2^i^	0.75 (2)	2.20 (2)	2.8967 (10)	154 (2)
C4—H4*A*⋯O1^ii^	0.990 (18)	2.350 (18)	3.3045 (11)	161.7 (13)
C10—H10*A*⋯O3^iii^	0.975 (16)	2.448 (14)	3.2012 (11)	133.8 (12)
C16—H16*B*⋯O1^iv^	0.953 (17)	2.565 (17)	3.4698 (12)	158.7 (13)
C15—H15*B*⋯*Cg*1	0.95 (2)	2.812 (19)	3.4494 (11)	125.3 (15)

Symmetry codes: (i) 
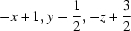
; (ii) 
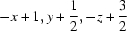
; (iii) 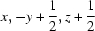; (iv) 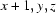.

**Fig. 1.**
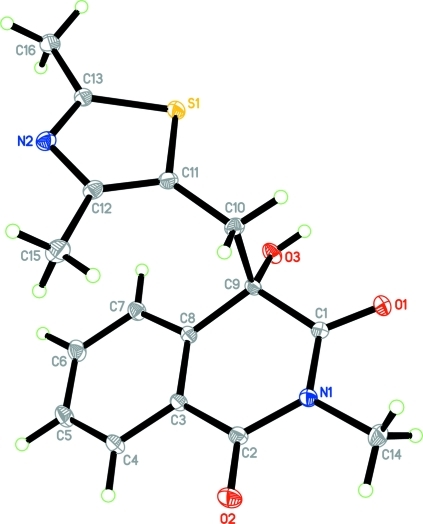
The asymmetric unit of the title compound, showing the atomic numbering scheme. Displacement ellipsoids are drawn at the 50% probability level.

**Fig. 2.**
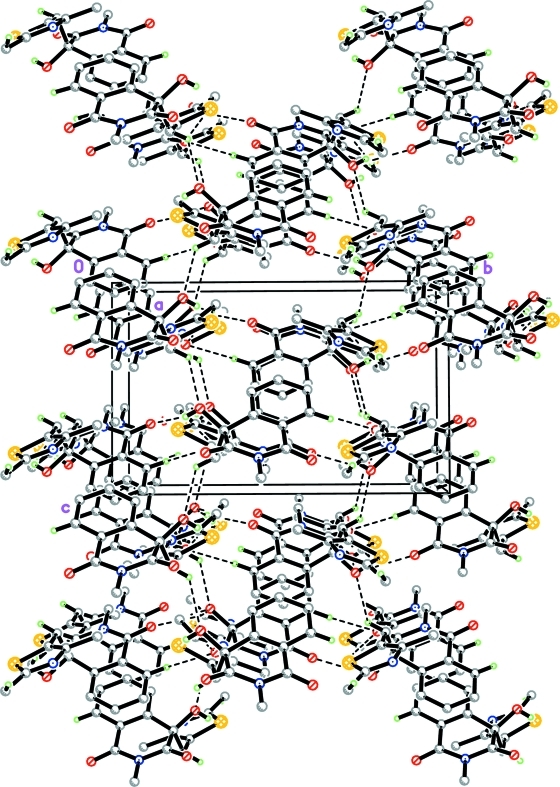
The crystal packing of the title compound, viewed along the a axis, showing the three-dimensional extended network. H atoms not involved in intermolecular interactions have been omitted for clarity.

## supplementary crystallographic information

### Comment

1,3,4(2*H*)-Isoquinolinetrione derivatives have a variety of biological activities and are synthetic precursors for many naturally occurring alkaloids (Hall *et al.*, 1994; Malamas & Hohman, 1994). The carbonyl group on C4 of isoquinoline-1,3,4-trione is an active site for photo-induced reactions with alkenes or other hydrogen donors to give photoaddition products such as oxetanes (Suau & Villatoro, 1994). Oxazole derivatives have been used as electron donor species in the Paternò-Büchi photocycloaddition with carbonyl groups (Griesbeck *et al.*, 2003). Other interesting photo-reactions such as the [4+4] photocycloadditions have also been reported on substituted oxazole with 9,10-phenanthraquinone and 1-acetylisatin (Zhang *et al.*, 2004). The crystal structure of *Z*-2-methyl-3'-phenyl-spiro[isoquinoline-4,2'-oxirane]-1,3-dione has been reported (Wang *et al.*, 2000). In view of the importance of the title compound as a typical H-abstracted product in photoreaction between carbonyl and thiazoles, the paper reports its crystal structure.

In the title isoquinolinedione derivative (Fig. 1), atom C9 is the chiral center. The piperidine ring (C1/N1/C2/C3/C8/C9) of the tetrahydroisoquinoline ring system adopts a distorted envelope conformation with atom C9 as the flap; the puckering amplitude Q = 0.2512 (9) Å, θ = 113.8 (2)° and φ = 291.0 (2)° (Cremer & Pople, 1975). The thiazol ring (C11/C12/N2/C13/S1) is essentially planar, with maximum deviation of 0.004 (1) Å at atom C11, and it inclines at a dihedral angle of 31.08 (3)° with the tetrahydroisoquinoline ring system. Bond lengths and angles are consistent to those observed in related isoquinoline-1,3-dione structures (Fun *et al.* 2010*a,b*; Zhang *et al.*, 2004).

In the crystal packing (Fig. 2), intermolecular O3—H1O3···O2, C4—H4A···O1, C10—H10A···O3 and C16—H16B···O1 hydrogen bonds (Table 1) link neighbouring molecules into a three-dimensional extended network. The crystal structure is further stabilized by intermolecular C15—H15B···*Cg*1 interactions (Table 1) involving the centroid of the C3–C8 benzene ring.

### Experimental

The title compound was obtained in the reaction between 1,3,4(2*H*)-isoquinolinetrione and 1,4,5-trimethyl thiazoles under photo-irradiation with light of wavelength > 400 nm. The compound was purified by flash column chromatography with ethyl acetate and petroleum ether as eluents. X-ray quality single crystals of the title compound were obtained by slow evaporation of solvents from the solution of the title compound in acetone and petroleum ether.

### Refinement

All the H atoms were located in a difference Fourier map and allowed to refine freely. Refined distances: C—H = 0.917 (16)–0.994 (15) Å.

### Crystal data

**Table:**

C_16_H_16_N_2_O_3_S	*F*(000) = 664
*M**_r_* = 316.37	*D*_x_ = 1.441 Mg m^−^^3^
Monoclinic, *P*2_1_/*c*	Mo *K*α radiation, λ = 0.71073 Å
Hall symbol: -P 2ybc	Cell parameters from 9887 reflections
*a* = 10.2424 (8) Å	θ = 2.5–35.1°
*b* = 15.0438 (13) Å	µ = 0.24 mm^−^^1^
*c* = 9.4786 (8) Å	*T* = 100 K
β = 92.839 (2)°	Block, colourless
*V* = 1458.7 (2) Å^3^	0.52 × 0.26 × 0.09 mm
*Z* = 4	

### Data collection

**Table:**

Bruker SMART APEX DUO CCD area-detector diffractometer	5260 independent reflections
Radiation source: fine-focus sealed tube	4752 reflections with *I* > 2σ(*I*)
graphite	*R*_int_ = 0.024
φ and ω scans	θ_max_ = 32.5°, θ_min_ = 2.7°
Absorption correction: multi-scan (*SADABS*; Bruker, 2009)	*h* = −15→15
*T*_min_ = 0.887, *T*_max_ = 0.979	*k* = −21→22
20742 measured reflections	*l* = −14→13

### Refinement

**Table:**

Refinement on *F*^2^	Primary atom site location: structure-invariant direct methods
Least-squares matrix: full	Secondary atom site location: difference Fourier map
*R*[*F*^2^ > 2σ(*F*^2^)] = 0.031	Hydrogen site location: inferred from neighbouring sites
*wR*(*F*^2^) = 0.108	All H-atom parameters refined
*S* = 1.14	*w* = 1/[σ^2^(*F*_o_^2^) + (0.0649*P*)^2^ + 0.2915*P*] where *P* = (*F*_o_^2^ + 2*F*_c_^2^)/3
5260 reflections	(Δ/σ)_max_ = 0.001
263 parameters	Δρ_max_ = 0.69 e Å^−^^3^
0 restraints	Δρ_min_ = −0.47 e Å^−^^3^

### Special details

**Table:**

Experimental. The crystal was placed in the cold stream of an Oxford Cryosystems Cobra open-flow nitrogen cryostat (Cosier & Glazer, 1986) operating at 100.0 (1)K.
Geometry. All esds (except the esd in the dihedral angle between two l.s. planes) are estimated using the full covariance matrix. The cell esds are taken into account individually in the estimation of esds in distances, angles and torsion angles; correlations between esds in cell parameters are only used when they are defined by crystal symmetry. An approximate (isotropic) treatment of cell esds is used for estimating esds involving l.s. planes.
Refinement. Refinement of F^2^ against ALL reflections. The weighted R-factor wR and goodness of fit S are based on F^2^, conventional R-factors R are based on F, with F set to zero for negative F^2^. The threshold expression of F^2^ > 2sigma(F^2^) is used only for calculating R-factors(gt) etc. and is not relevant to the choice of reflections for refinement. R-factors based on F^2^ are statistically about twice as large as those based on F, and R- factors based on ALL data will be even larger.

### Fractional atomic coordinates and isotropic or equivalent isotropic displacement parameters (Å^2^)

**Table:**

	*x*	*y*	*z*	*U*_iso_*/*U*_eq_	
S1	0.86697 (2)	0.201051 (14)	0.69225 (2)	0.01443 (7)	
O1	0.38478 (7)	0.29484 (4)	0.78648 (8)	0.01610 (14)	
O2	0.46407 (7)	0.59074 (5)	0.82383 (8)	0.01766 (14)	
O3	0.57054 (7)	0.27421 (4)	0.59312 (7)	0.01403 (13)	
N1	0.42666 (7)	0.44221 (5)	0.81117 (8)	0.01168 (13)	
N2	1.03523 (7)	0.32678 (5)	0.71641 (8)	0.01323 (14)	
C1	0.45580 (8)	0.35744 (5)	0.76386 (9)	0.01077 (14)	
C2	0.49293 (8)	0.51881 (6)	0.77347 (9)	0.01180 (14)	
C3	0.59432 (8)	0.50884 (5)	0.66814 (9)	0.01079 (14)	
C4	0.64725 (9)	0.58622 (6)	0.61172 (10)	0.01449 (16)	
C5	0.73988 (9)	0.57934 (6)	0.51012 (10)	0.01681 (17)	
C6	0.77833 (9)	0.49566 (6)	0.46357 (10)	0.01545 (16)	
C7	0.72519 (9)	0.41869 (6)	0.51860 (9)	0.01266 (15)	
C8	0.63326 (8)	0.42498 (5)	0.62248 (8)	0.01004 (14)	
C9	0.58517 (8)	0.34274 (5)	0.69351 (8)	0.00987 (14)	
C10	0.68361 (8)	0.31399 (6)	0.81709 (9)	0.01249 (15)	
C11	0.81935 (8)	0.29894 (5)	0.77079 (9)	0.01180 (15)	
C12	0.92179 (8)	0.35759 (6)	0.77307 (9)	0.01249 (15)	
C13	1.02070 (8)	0.24530 (6)	0.66982 (9)	0.01282 (15)	
C14	0.31069 (9)	0.45045 (6)	0.89534 (10)	0.01658 (17)	
C15	0.92066 (10)	0.45085 (7)	0.82696 (11)	0.01856 (18)	
C16	1.12481 (9)	0.19431 (6)	0.59986 (11)	0.01685 (17)	
H4A	0.6166 (16)	0.6451 (12)	0.6429 (18)	0.032 (4)*	
H5A	0.7767 (17)	0.6330 (12)	0.4682 (18)	0.032 (4)*	
H6A	0.8450 (15)	0.4910 (11)	0.3912 (16)	0.022 (4)*	
H7A	0.7529 (14)	0.3644 (11)	0.4837 (16)	0.022 (4)*	
H10A	0.6458 (14)	0.2612 (11)	0.8585 (15)	0.020 (3)*	
H10B	0.6861 (14)	0.3595 (10)	0.8887 (15)	0.018 (3)*	
H14A	0.2427 (16)	0.4203 (11)	0.8507 (16)	0.023 (4)*	
H14B	0.2864 (18)	0.5111 (13)	0.8938 (19)	0.038 (5)*	
H14C	0.3297 (17)	0.4255 (12)	0.9861 (18)	0.032 (4)*	
H15A	1.0023 (19)	0.4647 (13)	0.870 (2)	0.040 (5)*	
H15B	0.9089 (19)	0.4918 (15)	0.751 (2)	0.048 (5)*	
H15C	0.8533 (19)	0.4625 (13)	0.886 (2)	0.041 (5)*	
H16A	1.1181 (16)	0.2067 (11)	0.5008 (17)	0.027 (4)*	
H16B	1.2092 (17)	0.2095 (11)	0.6394 (18)	0.030 (4)*	
H16C	1.1129 (18)	0.1315 (14)	0.6097 (19)	0.037 (5)*	
H1O3	0.544 (2)	0.2340 (15)	0.629 (2)	0.043 (5)*	

### Atomic displacement parameters (Å^2^)

**Table:**

	*U*^11^	*U*^22^	*U*^33^	*U*^12^	*U*^13^	*U*^23^
S1	0.01309 (11)	0.00929 (11)	0.02129 (12)	0.00031 (6)	0.00483 (8)	0.00026 (7)
O1	0.0134 (3)	0.0105 (3)	0.0246 (3)	−0.0022 (2)	0.0038 (2)	0.0018 (2)
O2	0.0202 (3)	0.0099 (3)	0.0233 (3)	0.0007 (2)	0.0050 (3)	−0.0041 (2)
O3	0.0196 (3)	0.0086 (3)	0.0142 (3)	−0.0027 (2)	0.0042 (2)	−0.0027 (2)
N1	0.0116 (3)	0.0093 (3)	0.0144 (3)	−0.0001 (2)	0.0033 (2)	−0.0007 (2)
N2	0.0117 (3)	0.0124 (3)	0.0156 (3)	0.0014 (2)	0.0013 (2)	−0.0018 (2)
C1	0.0104 (3)	0.0092 (3)	0.0127 (3)	0.0004 (2)	0.0007 (2)	0.0003 (3)
C2	0.0123 (3)	0.0093 (3)	0.0138 (3)	0.0000 (3)	0.0000 (3)	−0.0005 (3)
C3	0.0114 (3)	0.0088 (3)	0.0121 (3)	−0.0007 (3)	0.0004 (3)	0.0008 (2)
C4	0.0160 (4)	0.0091 (3)	0.0184 (4)	−0.0016 (3)	0.0006 (3)	0.0020 (3)
C5	0.0181 (4)	0.0135 (4)	0.0189 (4)	−0.0033 (3)	0.0023 (3)	0.0048 (3)
C6	0.0163 (4)	0.0160 (4)	0.0143 (3)	−0.0022 (3)	0.0032 (3)	0.0034 (3)
C7	0.0141 (3)	0.0124 (3)	0.0116 (3)	−0.0008 (3)	0.0020 (3)	0.0007 (3)
C8	0.0110 (3)	0.0091 (3)	0.0101 (3)	−0.0009 (2)	0.0002 (2)	0.0009 (2)
C9	0.0111 (3)	0.0074 (3)	0.0111 (3)	−0.0002 (2)	0.0018 (2)	−0.0001 (2)
C10	0.0119 (3)	0.0139 (3)	0.0118 (3)	0.0028 (3)	0.0030 (3)	0.0027 (3)
C11	0.0119 (3)	0.0115 (3)	0.0121 (3)	0.0021 (3)	0.0020 (3)	0.0014 (2)
C12	0.0117 (3)	0.0128 (3)	0.0129 (3)	0.0020 (3)	0.0003 (3)	−0.0022 (3)
C13	0.0120 (3)	0.0113 (3)	0.0154 (3)	0.0010 (3)	0.0027 (3)	0.0005 (3)
C14	0.0144 (4)	0.0163 (4)	0.0197 (4)	0.0002 (3)	0.0075 (3)	−0.0016 (3)
C15	0.0156 (4)	0.0162 (4)	0.0237 (4)	0.0015 (3)	−0.0007 (3)	−0.0087 (3)
C16	0.0150 (4)	0.0126 (4)	0.0234 (4)	0.0022 (3)	0.0060 (3)	−0.0022 (3)

### Geometric parameters (Å, °)

**Table:**

S1—C11	1.7311 (9)	C6—H6A	0.994 (15)
S1—C13	1.7323 (9)	C7—C8	1.3988 (11)
O1—C1	1.2155 (10)	C7—H7A	0.931 (16)
O2—C2	1.2248 (10)	C8—C9	1.5031 (11)
O3—C9	1.4059 (10)	C9—C10	1.5681 (12)
O3—H1O3	0.75 (2)	C10—C11	1.4957 (12)
N1—C1	1.3891 (11)	C10—H10A	0.974 (16)
N1—C2	1.3930 (11)	C10—H10B	0.964 (15)
N1—C14	1.4685 (11)	C11—C12	1.3703 (12)
N2—C13	1.3089 (11)	C12—C15	1.4933 (13)
N2—C12	1.3839 (11)	C13—C16	1.4951 (12)
C1—C9	1.5287 (11)	C14—H14A	0.917 (16)
C2—C3	1.4834 (12)	C14—H14B	0.95 (2)
C3—C8	1.3984 (11)	C14—H14C	0.950 (17)
C3—C4	1.4012 (12)	C15—H15A	0.94 (2)
C4—C5	1.3888 (13)	C15—H15B	0.95 (2)
C4—H4A	0.990 (18)	C15—H15C	0.928 (19)
C5—C6	1.3970 (14)	C16—H16A	0.957 (16)
C5—H5A	0.983 (18)	C16—H16B	0.953 (18)
C6—C7	1.3919 (12)	C16—H16C	0.96 (2)
			
C11—S1—C13	90.17 (4)	C1—C9—C10	104.67 (6)
C9—O3—H1O3	108.5 (16)	C11—C10—C9	113.28 (7)
C1—N1—C2	124.16 (7)	C11—C10—H10A	112.9 (9)
C1—N1—C14	116.45 (7)	C9—C10—H10A	105.8 (9)
C2—N1—C14	119.14 (7)	C11—C10—H10B	108.8 (9)
C13—N2—C12	111.16 (7)	C9—C10—H10B	108.9 (9)
O1—C1—N1	120.95 (8)	H10A—C10—H10B	107.0 (13)
O1—C1—C9	120.27 (8)	C12—C11—C10	128.32 (8)
N1—C1—C9	118.58 (7)	C12—C11—S1	108.83 (6)
O2—C2—N1	120.06 (8)	C10—C11—S1	122.73 (7)
O2—C2—C3	122.83 (8)	C11—C12—N2	115.80 (8)
N1—C2—C3	117.08 (7)	C11—C12—C15	126.26 (8)
C8—C3—C4	120.65 (8)	N2—C12—C15	117.92 (8)
C8—C3—C2	121.30 (7)	N2—C13—C16	123.95 (8)
C4—C3—C2	118.02 (7)	N2—C13—S1	114.04 (6)
C5—C4—C3	119.55 (8)	C16—C13—S1	122.00 (7)
C5—C4—H4A	120.7 (10)	N1—C14—H14A	108.9 (10)
C3—C4—H4A	119.7 (10)	N1—C14—H14B	107.0 (11)
C4—C5—C6	119.94 (8)	H14A—C14—H14B	106.1 (15)
C4—C5—H5A	120.5 (10)	N1—C14—H14C	108.9 (10)
C6—C5—H5A	119.5 (10)	H14A—C14—H14C	109.9 (14)
C7—C6—C5	120.65 (8)	H14B—C14—H14C	115.9 (16)
C7—C6—H6A	119.7 (9)	C12—C15—H15A	109.7 (12)
C5—C6—H6A	119.7 (9)	C12—C15—H15B	110.7 (13)
C6—C7—C8	119.79 (8)	H15A—C15—H15B	105.4 (17)
C6—C7—H7A	117.7 (9)	C12—C15—H15C	113.7 (12)
C8—C7—H7A	122.5 (9)	H15A—C15—H15C	111.5 (16)
C3—C8—C7	119.41 (7)	H15B—C15—H15C	105.4 (16)
C3—C8—C9	119.94 (7)	C13—C16—H16A	108.5 (10)
C7—C8—C9	120.44 (7)	C13—C16—H16B	110.9 (10)
O3—C9—C8	109.07 (7)	H16A—C16—H16B	111.1 (14)
O3—C9—C1	109.61 (7)	C13—C16—H16C	111.4 (11)
C8—C9—C1	112.75 (7)	H16A—C16—H16C	106.5 (14)
O3—C9—C10	110.26 (7)	H16B—C16—H16C	108.4 (15)
C8—C9—C10	110.41 (7)		
			
C2—N1—C1—O1	−170.99 (8)	C7—C8—C9—C1	−160.42 (7)
C14—N1—C1—O1	3.16 (12)	C3—C8—C9—C10	−91.86 (9)
C2—N1—C1—C9	14.15 (12)	C7—C8—C9—C10	82.90 (9)
C14—N1—C1—C9	−171.71 (7)	O1—C1—C9—O3	35.47 (11)
C1—N1—C2—O2	−177.44 (8)	N1—C1—C9—O3	−149.63 (7)
C14—N1—C2—O2	8.56 (13)	O1—C1—C9—C8	157.17 (8)
C1—N1—C2—C3	4.55 (12)	N1—C1—C9—C8	−27.93 (10)
C14—N1—C2—C3	−169.45 (8)	O1—C1—C9—C10	−82.79 (9)
O2—C2—C3—C8	174.08 (8)	N1—C1—C9—C10	92.12 (9)
N1—C2—C3—C8	−7.97 (12)	O3—C9—C10—C11	64.06 (9)
O2—C2—C3—C4	−8.02 (13)	C8—C9—C10—C11	−56.54 (9)
N1—C2—C3—C4	169.93 (8)	C1—C9—C10—C11	−178.13 (7)
C8—C3—C4—C5	−0.41 (13)	C9—C10—C11—C12	94.19 (11)
C2—C3—C4—C5	−178.32 (8)	C9—C10—C11—S1	−81.38 (9)
C3—C4—C5—C6	0.84 (14)	C13—S1—C11—C12	0.55 (7)
C4—C5—C6—C7	−0.35 (14)	C13—S1—C11—C10	176.87 (7)
C5—C6—C7—C8	−0.59 (13)	C10—C11—C12—N2	−176.72 (8)
C4—C3—C8—C7	−0.53 (12)	S1—C11—C12—N2	−0.66 (10)
C2—C3—C8—C7	177.31 (7)	C10—C11—C12—C15	1.63 (15)
C4—C3—C8—C9	174.28 (8)	S1—C11—C12—C15	177.69 (8)
C2—C3—C8—C9	−7.87 (12)	C13—N2—C12—C11	0.41 (11)
C6—C7—C8—C3	1.02 (13)	C13—N2—C12—C15	−178.08 (8)
C6—C7—C8—C9	−173.77 (8)	C12—N2—C13—C16	178.59 (8)
C3—C8—C9—O3	146.83 (8)	C12—N2—C13—S1	0.04 (10)
C7—C8—C9—O3	−38.41 (10)	C11—S1—C13—N2	−0.35 (7)
C3—C8—C9—C1	24.83 (10)	C11—S1—C13—C16	−178.93 (8)

### Hydrogen-bond geometry (Å, °)

**Table:**

Cg1 is the centroid of C3–C8 benzene ring.

**Table:**

*D*—H···*A*	*D*—H	H···*A*	*D*···*A*	*D*—H···*A*
O3—H1O3···O2^i^	0.75 (2)	2.20 (2)	2.8967 (10)	154 (2)
C4—H4A···O1^ii^	0.990 (18)	2.350 (18)	3.3045 (11)	161.7 (13)
C10—H10A···O3^iii^	0.975 (16)	2.448 (14)	3.2012 (11)	133.8 (12)
C16—H16B···O1^iv^	0.953 (17)	2.565 (17)	3.4698 (12)	158.7 (13)
C15—H15B···Cg1	0.95 (2)	2.812 (19)	3.4494 (11)	125.3 (15)

Symmetry codes: (i) −*x*+1, *y*−1/2, −*z*+3/2; (ii) −*x*+1, *y*+1/2, −*z*+3/2; (iii) *x*, −*y*+1/2, *z*+1/2; (iv) *x*+1, *y*, *z*.
